# Acknowledgement to Reviewers of *JCM* in 2018

**DOI:** 10.3390/jcm8010058

**Published:** 2019-01-08

**Authors:** JCM Editorial Office

**Affiliations:** MDPI, St. Alban-Anlage 66, 4052 Basel, Switzerland; jcm@mdpi.com

Rigorous peer-review is the corner-stone of high-quality academic publishing. The editorial team greatly appreciates the reviewers who contributed their knowledge and expertise to the journal’s editorial process over the past 12 months. In 2018, a total of 588 papers were published in the journal, with a median time to first decision of 15 days and a median time to publication of 31 days. The editors would like to express their sincere gratitude to the following reviewers for their cooperation and dedication in 2018:
Aagaard, Niels K.Häring, Hans-UlrichPerkisas, StanyAbbas-Aghababazadeh, FarnooshHaron, MonaPerloff, Michael D.Abdul Salim, SohailHartley, CarolinePersani, LucaAbe, MasanoriHartman, John D.Peter, LukášAbete, PasqualeHartmann, HagenPhillips, Mary E.Abuzaid, AhmedHautz, TheresaPiepmeier, AaronAddy, CharlotteHeads, Angela M.Pina, MelanieAhmed, NaseerHecker, AndreasPlasqui, GuyAiragnes, GuillaumeHedberg, PärPolak, SebastianAkamizu, TakashiHedhammar, MyPolasek, Thomas M.Alakhras, MaramHeldner, MirjamPoldermans, DonAl-Bahrani, RedaHelliwell, Philip S.Polguj, MichalAlbert, StewartHeo, Dae SeogPoli, GiuseppeAlesi, MariannaHes, Frederik J.Pomeroy, JeremyAloisi, Anna M.Hess, JochenPontrelli, PaolaAl-Samkari, HannyHeyn, PatriciaPostuła, MarekAlvarez-Llamas, GloriaHicks, AlexanderPreciado, DiegoAmmer, KurtHideaki, SoyaPrêle, CeciliaAmole, MorolakeHill, AileenPrior, Steven J.Anadol, RemziHilton, ClaudiaPriyamvada, LalitaAnderson, CraigHirahara, NoriyukiQuencer, KeithAndersson, KarlHochberg, IritQuigley, EamonnAnsani, LuciaHoff, PaulaR. Shaker, MohammedAntunes, HugoHoffman, RobertRachek, Lyudmila I.Anurag, MeenakshiHomko, Carol J.Rago, AnnaAraújo, João RicardoHoppe, Liesa KatharinaRall, Glenn F.Arcila, Maria E.Hori, HikaruRambhatla, AmarnathArentson-Lantz, EmilyHoyer, Erik H.Ramot, YuvalArmando, StefanatiHu, BohuaRasuli, PasteurArnaout, BachaarHuang, LongshuangRaw, Rachael K.Artioli, GraziaHuang, Shiang-FenReichert, David E.Artunc, FerruhHuang, Shih-YiReimers, Anne K.Arveschoug, Anne K.Hughson, Michael D.Reiner, Anne S.Asatryan, BabkenHuh, Jin WonReiser, JochenAshton, JamesHwang, JungyunReisfeld, SharonAssenat, EricHyman, James M.Remuzzi, GiuseppeAvadhanula, VasanthiIgarashi, KazueiReschke, Millard F.Aveline, ChristopheIglesias, Juan F.Reynolds, Katharine C.Azuma, ToshifumiIgloi, KingaReznikov, Leah R.Backer, Daniel DeIliou, Marie C.Richards, Jeremy B.Badlissi, FadiImai, TakaoRimaitis, KęstutisBalasubramanian, GaneshImfeld, PatrickRivas, Carol A.Ballesteros, SoledadIngenbleek, YvesRivera, EleanorBalouch, SaraIno, YasushiRizvi, Ali A.Banach, MaciejInokuchi, WataruRizzo, GiuseppeBancos, IrinaIorio, Anna L.Robert, BattatBang, Marie-LouiseIorio, RaffaeleRoberts, Drucilla J.Baniahmad, AriaItamar, RazRobinson, EdwardBanyasz, Alissa M.Ito, ShigekiRobinson, PhilipBaracos, VickieIzumi, KoujiRoeser, MarkBaragetti, AndreaJaberi, AalaRoilides, EmmanuelBaran, RobertJacobs, DavidRojas-Peña, AlvaroBarchetta, IlariaJaekel, JuliaRomo, JesusBarichello, TatianaJaimes, Edgar A.Ronco, PierreBarranco Ruiz, YairaJain, HanaRonellenfitsch, UlrichBarratt, ShaneyJalava, KatriRoper, Randall J.Barreto, Erin F.Jayant, KumarRosaly, Correa-de-AraujoBarton, Matthew J.Johnson, StuartRosenfield, MarkBastian, MaxJorsäter-Blomgren, KerstinRosenzweig, DerekBatinic-Haberle, InesJost, Gregory F.Rossi, ElioBauer, CorinnaJuhasz, BelaRothe, HansjörgBauer, Stuart B.Jules, KevinRouse, RodneyBaydur, AhmetJúlíusson, Pétur BenediktRoviello, GiandomenicoBeaumont, RobinKadambi, Pradeep V.Rudrappa, MohanBeckmann, ManfredKakinuma, YoshihikoRuppen, WilhelmBeer, Ambros J.Kalfert, DavidRutegård, MartinBeeri, Michal S.Kallonen, TeemuRzońca, EwaBehrendt, Christian A.Kalra, SanjogSacerdote, PaolaBeisswenger, ChristophKalwat, Michael A.Saintigny, PierreBejjanki, HariniKamijo-Ikemori, AtsukoSakhaee, KhashayarBerger, MetteKamiya, HidekiSakurada, TsutomuBermejo-Martin, Jesús F.Kampmann, UllaSalahudeen, Mohammed SajiBernat, James L.Kang, InhaeSaleh, TareqBernstein, Hans-GertKantrowitz, JoshuaSalgarelli, Attilio C.Berveiller, PaulKanzow, PhilippSalinas, JenniferBhutta, MahmoodKarakochuk, CrystalSalmasi, AmiraliBijlsma, Maarten J.Karan, RadmilaSalminen, PaulinaBikov, AndrásKarlsson, HakanSambri, VittorioBirchard, ThaddeusKaskel, FrederickSamuel, StuartBiruete, AnnabelKaushik, AshleshaSánchez, MikelBishwajit, GhoseKawulok, MichalSancho, JaimeBoentert, MatthiasKazemi-Bajestani, Seyyed Mohammad RezaSandblom, GabrielBoezeman, Edwin J.Kazuhiko, TsuruyaSands, Tristan T.Borghi, ClaudioKeane, DavidSantos, LuisBosman, GielKeane, KevinSantosa, HendrikBottiger, YlvaKeenan, MichaelSapey, ElizabethBowling, AprilKeklikoglou, IoannaSasahira, TomonoriBraat, Andries E.Kelz, MaxSaulacic, NikolaBradley, Elyse C.Kennedy, Mary-ClaireSavarino, VincenzoBrand, SergeKennedy, NoreleeSavastano, SilviaBrazo, PerrineKern, BenSchattman, Glenn L.Briassoulis, GeorgeKhoury, SamarSchernthaner, Gerit-HolgerBrietzke, Scott E.Kidd, Timothy J.Scherrer, Jeffrey F.Brochiero, EmmanuelleKim, BhumsooSchiano, ConcettaBrown, Joshua D.Kim, Jennifer A.Schob, StefanBruins Slot, KarstenKindermann, HaraldSchreckenberger, MathiasBryniarski, KrzysztofKinoshita, AkiyoshiSchumacher, UdoBuckley, NicholasKirwan, ChrisSearle, Amelia K.Buell, Thomas J.Knohl, Stephen J.Sebastian, LudygaBujan-Rivas, SegundoKnott, FionaSelva-O’Callaghan, AlbertBukhari, Marwan A.S.Kobler, JamesSenesi, PamelaBulum, TomislavKodama, DaisukeSessa, AurelioBurjek, Nicholas E.Koga, FumitakaShapiro, Charles L.Burkard, ThiloKolen, Angela M.Sharma, AdityaBurr, LucyKonialis, ChristopherSharma, IshaBush, DouglasKonstantinidou, Maria K.Sharma, PawanBuxi, DilpreetKoshiyama, MasafumiSharma, RitinCadario, FrancescoKosmidou, IoannaSharpe, LouiseCaironi, PietroKotsaki, AntigoniShaw, George J.Calabro, Rocco SalvatoreKrafft, Christoph​Sherratt, SimonCalle Rubio, MyriamKrause, IritShi, YiCammu, GuyKroiss, AlexanderShiga, KiyotoCappariello, AlfredoKropp, MartinaShirotsuki, KentaroCarella, Angelo M.Kruger, Tillmann H.C.Shiu, Yan-TingCaretti, AnnaKrupinski, ElizabethShortliffe, Linda M. DairikiCarulli, ChristianKukshal, VandnaShulman, AdrianCasans Francés, RubénKullberg, Bart-JanShultz, SandyCasarini, LivioKumar, AmitShunya, UchidaCasella, MichelaKumar, JayantSigvardsdotter, ErikaCastagnini, FrancescoKünzel, JulianSilbert, BenjaminCasucci, GerardoKuperman, Ethan F.Simka, MarianCazzador, DiegoKuźma-Kozakiewicz, MagdalenaSingh, FavilCetin, IreneKuzmiene, LoretaSingh, NaveenChacón-Borrego, FátimaKwiatkowski, DavidSinjari, BrunaChamaria, SurbhiLaMarca, BabetteSisley, StephanieChang, Ke-VinLangabeer, James R.Sisti, GiovanniChappel, EricLappin, TerrySitia, GiovanniChehroudi, BabakLarange, AlexandreSivapalan, PradeeshChen, Kuo-HuLeach, Steven T.Skaug, BrianChen, Yun-JuLee, MinsunSkinner, Jonathan R.Chhabra, SaurabhLee, TaikjinSkoien, RichardChiang, Tai-AnLeger, LucSlankamenac, KsenijaChiappori, Alberto A.Lehrer, PaulSmalling, Richard W.Childs, Jessie T.Lemoine, Marie-Eve LemoineSmith, Judith A.Chin, PaulLempereur, MathieuSmith, PatrickChiu, Yu-HanLenders, JacquesSöderström, LisaChmurzyńska, AgataLeonetti, CarloSoiza, Roy L.Chou, AngelaLewald, HeidrunSong, Yin-ChenChoudhury, DevasmitaLi, Chia-JungSood, Manish M.Chue, Pierre S.Li, Hong-ZheSorensen, CarlCicero, ArrigoLi, Shu-ZhaoSoto-Pérez, FelipeCitterio, DavideLiabeuf, SophieSpetea, MarianaClark, EdwardLiamis, GeorgeSpradley, Frank T.Clouston, Sean A.P.Liao, Ai-HoSquires, PaulCoccheri, SergioLicari, AmeliaStančin, SaraCoffey, Robert J.Lichtenstein, Mia B.Stansfield, KirstieCohen, Steven M.Liehn, Elisa A.Starostik, PetrCooper, SimonLillehoj, ErikSteiner, Laurie A.Corr, PhilipLin, Chih-PengStepien, Karolina M.Correale, MicheleLin, Rui-TingStodt, BenjaminCortese, AntonioLindner, PhilipStoffregen, ThomasCortese, FrancescaLingappan, KrithikaStringhini, SilviaCosta, FrancesoLink, AlexanderStunes, Astrid K.Cote, PatriceLipnicki, DarrenSturiale, Carmelo L.Coulter, Jonathan A.Lippert-Rasmussen, KasperStylianou, KonstantinosCrescioli, ClaraLipson, Michael J.Sukhal, ShashvatCrisafulli, ErnestoLittlefield, AndrewSullivan, Mark D.Crosbie-Watson, RachelleLivraghi-Butrico, AlessandraSun, Zhong-HuaCrowley, JenniferLogan, SreemathiSuto, TakahitoCrowley Jr., William F.Lomero, Marta GómaraSuzuki, TakeshiCunha, RodrigoLong, ElizabethSvensson, JannetCuspidi, CesareLopez-Campos, Jose LuisSzeliga, JacekCybulski, MateuszLópez-López, JoséSztal, TamarDamalanka, VishnuLopomo, Nicola F.Szuhany, Kristin L.Danner, Simon M.Louch, William E.Tagliaferri, SaraDarcy, JustinLuo, Hua-ChengTakahashi, YusukeDasari, SurendraLupien, AndréanneTakaya, JunjiDatta, PoulamiLutz, WolfgangTakebayashi, ShigeoDaubert, Diane M.Lynch, ChristopherTakiyama, YumiDe Hair, MariaMa, Xing-JunTaniguchi, HiroyaDe La Iglesia-Garcia, DanielMaas, RenkeTanner, BoboDe Lucia, OrazioMacMillan, FreyaTariq, SarahDe Nicola, LucaMagenes, GiovanniTarragon Cros, ErnestoDe Pinto, MarioMagni, PaoloTedla, Yacob G.Dedy, Nicolas J.Malik, AfshanTeede, Helena J.Degens, HansMalone, Daniel C.Teles, AlissonDel Bono, ValerioMamas, ThaliaTennant, Matthew T.S.Demidem, AichaMañago, Mark M.Teques, PedroDeraco, MarcelloMandigout, StéphaneThamilselvan, SivagnanamDeschamps, JeanMang, Thomas S.Thevarajan, IraniDetloff, Megan RyanMännikkö, MinnaThiersch, MarkusDetti, LauraMansky, Kim C.Thomas, Lucy C.D’Ettorre, GabrieleMantadakis, ElpisThomas, Rebecca L.Dev Jayant, RahulManzano, LuisThompson, WadeDevy, ZismanManzano, RaquelThunnissen, ErikDhanekula, Raja KoteswarMarc, MiravitllesThuzar, MoeDi Mauro, MicheleMargulis, Andrea V.Ticconi, CarloDi Stefano, AntonioMarozas, VaidotasTidbury, Laurence P.Dias, Irundika H.K.Marra, PaoloTieland, MichaelDidehbani, NyazMarteau, PhilippeTiller, DanielDiMeglio, Linda A.Martínez-Pinilla, EvaTimman, ReinierDionisi, FrancescoMartinez-Salgado, CarlosTitus, Mark A.Dissanayake, HasthiMarverti, GaetanoTjernberg, IvarDjukanović, LjubicaMassa, HoraceTokodai, KazuakiDonadio, CarloMatsunaga, KazuhisaToma, ClaudioDonofry, Shannon D.Matsuzaki, JunichiTonga, KatrinaDontje, ManonMattace-Raso, Francesco U.S.Torgerson, Paul R.Doulias, Paschalis-ThomasMattan, Bradley D.Torres, Alcy R.Dowland, Samson N.Matteoli, SaraTosur, MustafaDruey, Kirk M.Mattsson, JanetTõugu, VelloDuann, Jeng-RenMcGregor, GordonTouraine, PhilippeDublin, SaschaMcHugh, NeilTownsend, EleanorDudakova, LubicaMcKenna, Peter E.Trachana, VarvaraDullaart, Robin P.F.McLoney, Eric D.Treasure, TomDumitrascu, DanMcPhee, JamieTrimarco, BrunoDumitrascu, TraianMcWilliams, Daniel F.Trinkmann, FrederikDusso, AdrianaMearelli, FilippoTrino, StefaniaDutta, SunilMeijers, BjörnTrummer, OliviaDwidmuthe, SamirMekontso Dessap, ArmandTsuruda, ToshihiroEaton, WilliamMelkani, GirishTunér, JanEkúndayò, Olúgbémiga T.Mellemkjaer, LeneTurner, Raymond S.Elson, DanielMercieri, MarcoTvedt, Tor Henrik AndersonEmilsson, LouiseMete, OzgurTwomey, NiallEnomoto, HirayukiMichálek, PavelUfholz, Kelsey E.Esteve-Puig, RosauraMigliore, FedericoUngaro, PaolaEvangelista, LauraMilanaik, Ruth LynnValomon, AmandineEveraert, KarelMills, Jesse N.Van Ballegooijen, HanneFacchin, FedericaMilone, MarcoVan Den Helder, JantineFacchinetti, FabioMin, SedongVan Hise, Nicholas W.Farber, Harrison W.Misra, StutiVan Rompay, Koen K.A.Farrell, Stephen W.Mitchell, LanaVan Ryn, JoanneFawkner, Samantha G.Mittal, GayatriVan Weerden, WytskeFeldman, Klara R.Mizuta, KentaroVarga, EszterFerrie, SuzieMocellin, SimoneVashisht, RohitFinn, Aloke V.Mochizuki, NaokiVassallo, RobertFischli, StefanMoen, Aina E.F.Vassiliou, Aliki G.Fliedner, StephanieMohammed, ManalVassiliou, Leandros-VassiliosFloros, GeorgiosMolins, Claudia R.Vaucher, PaulForget, PatriceMollace, VincenzoVaz, PaulaFormenti, FedericoMontrucchio, GiuseppeVehkaoja, AnttiFortunato, OrazioMorata, LauraVerguts, JasperFragkos, KonstantinosMorell, FerranVervoort, TineFrancesco, LocatelliMori, HirotadaVetter, Douglas E.Francois, PatriceMori, KiyoshiVicent, JesusFrankova, SonaMorres, IoannisViglianti, Elizabeth M.Franzè, AnnamariaMoustakas, AristidisVillani, AnthonyFraser, Steve F.Müller-Myhsok, BertramVilleneuve, LaurentFredriksson-Larsson, UllaMurakoshi, NobuyukiVitacca, MicheleFrisk, GunMurphy, Miles A.Vogel, ErinFrohman, MichaelMyers, MarkVoges, IngaFrykholm, PeterNachabé, Rami A.Von Lewinski, DirkFuelesdi, BelaNagpal, SeemaVrana, DavidFujita, KoheiNagulapalli Venkata, KalyanVukmirovic, MilicaFülöp, TiborNaka, HiroakiWaalen, JillFumagalli, FrancescaNapoli, AngelaWada, TomohitoFürnsinn, ClemensNaranjo-Hernández, DavidWang, Henry E.Gaitskell, KeziaNardi, PaoloWang, Peng-HuiGalbán, Craig J.Naruse, MitsuhideWang, Shao-MengGama, VivianNatarajan, ArutselvanWarburton, Darren E.R.Gandhi, Shashank V.Nathan, Cherie-Ann O.Ward, Roberta J.García Vela, José A.Nauck, MichaelWardle, JonGarg, LohitNeri, MatteoWargovich, MichaelGatla, HimavanthNestor, CaseyWasan, EllenGaudino, MarioNewell-Fugate, Annie E.Watanabe, AtsuyukiGeisler, CorinnaNicholas, Susanne B.Wedi, EdrisGentilini, FabioNicola, FuscoWeis, SebastianGhisletta, PaoloNicolau, DavidWenning, LeslieGholami, SepidehNicoletti, FerdinandoWerner, Gabriela G.Giaconi, Jo Ann A.Nierkens, StefanWesthoff-Bleck, MechthildGiannoni, PatriziaNiikura, RyotaWhelan, Fiona J.Gifford, PaulNikoletou, DimitraWhite, BeckyGillies, GlendaNishikawa, TetsuoWienbergen, HarmGitelman, Stephen E.Niyazov, Dmitriy M.Wiersinga, Willem J.Giulivi, CeciliaNoiri, EiseiWierzbicka, ElzbietaGiusti, Anna M.Nomellini, VanessaWillis, ErinGlasoe, Ward M.Nouri, AriaWillis, Gareth R.Glueck, Charles J.Nowak, Elizabeth C.Wilsey, MichaelGluud, LiseNowrouzi-Kia, BehdinWollin, DanielGnambs, TimoNugter, AnnetWong, Yon-CheongGnanamony, ManuNunez, Desmond A.Woroniecki, Robert P.Gold, Jason S.Nurwidya, FarizWright, EricGolding, JeanNyende, HawaWright, LauraGoldsmith, DavidOancea, CristianWu, Chih-JenGolestaneh, LadanO’Neal, HollisWu, Tong-ZhiGoodman, AnnekathrynOoi, KennethWu, Wen-ChihGoodyear, VictoriaØrngreen, Mette CathrineXiang, Hui-YunGordon, Eliza L.Ortega-Loayza, AlexXu, Da-ZhongGorelik, OlegOrtiz Arduan, AlbertoXu, Jun-WangGorringe, KylieOudman, ErikXu, Qin-ZiGoulielmos, George N.Ozen, GulsenXue, XiangGouvernet, BricePace, FabioYamada, TetsujiGrabowski, MarcinPadala, Kalpana P.Yamaoka, YoshioGråstén, ArtoPadmanabhan, JagannathYang, Eun JooGrattagliano, IgnazioPaduch, RomanYang, Kwang IkGraudins, Linda V.Pagel, James F.Yang, Wei-ShanGraves, Jennifer S.Paine, AnantaYang, Zheng-YuGreupink, RickPalladino, RaffaeleYavuz, MetinGrimaldi, DomenicoPan, Zhu-JunYavuz, SuleGris, Jean C.Panahiazar, MaryamYazawa, TakashiGrond-Ginsbach, CasparPapadopoulou, AlexandraYébenes, Juan C.Guedeney, PaulPaparella, DomenicoYeung, EugeneGugatschka, MarkusPapazoglou, KonstantinosYevzlin, Alexander S.Guru, PramodPark, Kwang B.Yin, JunHackett, GeoffParsons, BarbaraYokota, TomoyaHadjadj, SamyPascual, JulioYokoyama, HisayoHafer, CarstenPasquel, FranciscoYu, Guo-QinHage, CamillaPastori, DanieleYu, Min-ZhongHagner-Derengowska, MagdalenaPatron, Roberto L.Yuan, Li-YunHall, Shana A.Patschan, OliverYuzawa, YukioHall, Stephanie E.Pavarin, Raimondo M.Zaccardi, FrancescoHamdy, Reggie C.Pawlitzki, MarcZach, SimaHaneke, EckartPedergnana, VincentZakaria, DalaHannafon, BethanyPedro, Maria T.Zervos, Emmanuel E.Hans, GuyPeinado, Juan R.Zhang, Ling-JuanHansen, Solveig LenaPeitsidis, PanagiotisZhang, Meng-LiangHanudel, MarkPellicori, PierpaoloZhang, Qiu-YangHarari, SergioPeng, RobertZhao, WeiHarati, AliPerego, CarlaZoeller, ThomasHarauz, GeorgePérez-López, AlbertoZomer, Tizza P.Harcourt, BrookePérez-Vázquez, Paz


